# Beyond the seizure: the role of post-event observation in febrile seizure management

**DOI:** 10.1007/s00431-025-06659-8

**Published:** 2025-11-25

**Authors:** Gidon Test, Ana Luiza Zigman, Doron Pasternak, Dennis Scolnik, Vered Wiesel, Yousef Abu El Hasan, Remah Yousef, Oren Tavor, Inbal Kestenbom, Or Kaplan

**Affiliations:** 1 Pediatric Emergency Department, Soroka University Medical Center, Beer-Sheva, Israel; 2 Faculty of Health Sciences, Ben-Gurion University of the Negev, Beer-Sheva, Israel; 3 Clinical Research Center, Soroka University Medical Center, Beer-Sheva, Israel; 4 Division of Pediatric Emergency Medicine, Department of Pediatrics, The Hospital for Sick Children, University of Toronto, Toronto, Canada ON; 5 Pediatric Department D, Soroka University Medical Center, Beer Sheva, Israel

**Keywords:** Febrile, Convulsion, Observation, Recurrence

## Abstract

Simple febrile seizures (SFS) are a common cause of pediatric emergency department (PED) visits. Guidelines discourage extensive workup for children presenting with SFS, but there is little consensus on whether, or how long, to observe these patients. The objective of this study is to identify clinical, demographic, and historical factors associated with short-term seizure recurrence which might merit an observational period in children who have suffered a febrile seizure. This retrospective cohort study included children aged 12 months to 5 years who presented to the PED with a single generalized seizure in the setting of fever ≥ 38 °C. Current hospital policy recommends a 6-h period of observation in the PED for all such children, and admission for those who undergo a second seizure. Statistical analyses included comparisons between children with and without seizure recurrence, and logistic regression to identify predictors of hospitalization. Of 1496 children screened, 1404 met inclusion criteria. Seizure recurrence occurred in 27 (1.9%) of patients—all within 3 h of presentation. None of the following factors predicted seizure recurrence: gender, ethnic group, family history of febrile seizures, prior SFS, duration of fever before seizure, or peak fever. *Conclusion*: The short-term recurrence of febrile seizures was low (1.9%) and occurred within hours of the index seizure. Since the recurrence rate was low, and considering the benign outcomes of SFS, an observation period may not be warranted. Children who are stable and have regained neurological function may be considered for early discharge with appropriate caregiver education and follow-up, as already practiced in some centers.

**Table:** 

**What is Known:** •* Simple febrile seizures are frequent and often benign occurrences in children.* • *Guidelines discourage extensive tests but omit specific observation protocols.* **What is New:** • *Seizure recurrence after simple febrile seizures is very low and usually occurs within the first 3 h after the initial seizure.* • *After a simple febrile seizure, children may be considered for early discharge once they return to baseline function, without the need for an extended observation period.*

**What is Known:**

•* Simple febrile seizures are frequent and often benign occurrences in children.*

• *Guidelines discourage extensive tests but omit specific observation protocols.*

**What is New:**

• *Seizure recurrence after simple febrile seizures is very low and usually occurs within the first 3 h after the initial seizure.*

• *After a simple febrile seizure, children may be considered for early discharge once they return to baseline function, without the need for an extended observation period.*

## Introduction

Seizures account for 2–3.2% [1, 2] of pediatric emergency department (PED) visits, with febrile seizures comprising 28–53% of these cases. *Simple* febrile seizures (SFS), one of the most common neurological events in childhood, occur in 2–5% of children between 6 months and 5 years of age [3] account for 68% of all febrile seizures [4], and, by definition, occur in children who are otherwise neurologically healthy, i.e., no history of developmental delay or prior brain injury such as prematurity-related intraventricular hemorrhage, and with no other neurological disorders. SFS thus constitute a substantial portion of PED visits worldwide, posing distinct challenges for healthcare providers and exerting a strain on emergency care resources [5, 6]. Prediction of seizure recurrence and appropriate management strategies remain significant topics of interest and debate.

The definition of SFS excludes instances where seizure duration exceeds 15 min, the seizure is focal and if there is > 1 event within 24 h [3]. When a febrile seizure recurs within 24 h of the index seizure, the term *complex* febrile seizure is applied and, since there is an increased possibility of future afebrile seizures in such patients, they require different discharge decisions [7–9]. Identifying factors associated with recurrence of seizures within 24 h of an index febrile seizure would enhance management, risk stratification, and parental education [10, 11]. Such studies have been attempted, but often included mixed populations such as children with both simple and complex febrile seizures [12], children with pre-existing background diseases [13], and differing methods of follow-up [14]. Children from differing ethnic and population groups also experience different recurrence rates [15], which may, in turn, influence the duration of recommended observation periods [16]. Some studies have suggested that high temperatures at seizure onset and/or viral etiologies may predispose to recurrence of seizures within the first 24 h [17], and vigorous treatment of fever has been reported to assist in reducing the risk of seizure recurrence [18, 19].

The American Academy of Pediatrics advises against routine neuroimaging or laboratory evaluation [3]. The use of anti-epileptics is discouraged [3] and the treatment of fever management and underlying illness is encouraged [3, 5, 12]; however, the issue of observation is not mentioned. Some institutions do not practice any observation period following a SFS and discharge children home once neurological examination is normal. Others, such as our institution, practice a 6-h observation period, as recommended for children experiencing non-febrile seizures [20, 21]. Since this latter practice includes children with first time epileptic seizures, known neurological disease, and focal and complex seizures [20, 21], it may not be an optimal recommendation for children with febrile seizures.

The absence of standardized guidelines regarding the need for, and duration of, an observation period following a febrile seizure, and the management that should be undertaken during such a time period, underscores the need for more research evaluating current practices [22]. Since our institution currently observes children for 6 h following a febrile seizure, we have a large population of children in whom we were able to examine the role of specific historical, clinical, and demographic factors and treatments on seizure recurrence in a relatively uniform cohort of children.

## Methods

The electronic medical charts of children admitted to the PED of the Soroka Medical Center, Beersheba, Israel, during the 7 years from 2015 to 2022 inclusive with a febrile seizure were examined in a retrospective cohort study. Soroka University Medical Center serves as the sole pediatric emergency facility for the Negev region of southern Israel, covering a diverse urban and rural population. The following data were extracted: demographic information, historical features including details of prior seizures, clinical presentation including timing of fever onset, fever status at admission, maximum recorded temperature, timing of seizure, seizure duration, therapeutic interventions such as administration of antipyretics in the PED and at home, clinical parameters including weight, laboratory results, treatment provided, observation time, and final disposition. The category “Jewish” in this study reflects a sociocultural group as defined by national demographic reporting in Israel. This group includes individuals with diverse ancestral origins from Europe, the Middle East, North Africa, and the Americas, and should not be interpreted as genetically uniform. Family history was defined as having a first degree relative who suffered a febrile convulsion. Ours is the only hospital in our geographic area; thus, readmission rates were used to establish outcomes.

Our institutional protocol recommends a 6-h observation period for all children presenting with febrile seizures. However, clinical decision-making ultimately determines actual discharge timing based on factors including neurological recovery, identification of fever source, parental comfort level, and clinical judgment.

The study was approved by the Institutional Review Board of Soroka Medical Center.

### Inclusion criteria

1. ICD-9 diagnosis of febrile seizure recorded in the electronic medical chart.
2. A single, generalized tonic–clonic seizure, lasting < 15 min, associated with fever.
  - Fever was defined as at least one temperature measurement ≥ 38 °C during the seizure or within the 24 h immediately preceding or following the seizure. (Although the definition of SFS mandates the absence of an additional seizure during the same febrile illness, this detail was, by definition, not known at the time that the study was performed since patients were not observed until full resolution of the febrile episode, but only for the first 6 h after the febrile seizure.)
  - Normal neurological examination at presentation and/or return to normal neurological baseline during the observation period.
  - No history of developmental delay, prior brain injury, e.g., prematurity-related intraventricular hemorrhage, or any chronic neurological disorder.
3. Age 12 months to 5 years. This cutoff was selected since our hospital policy typically mandates that children younger than 12 months be hospitalized rather than undergo observation in the PED.

### Exclusion criteria

1. Age < 12 months or > 5 years.
2. Any known neurological condition or comorbidities that could explain or trigger the seizure episode.
3. Persistent neurological abnormalities.
4.  > 1 febrile seizure prior to arrival.
5. Focal seizure.
6. Seizure duration > 15 min.
7. Insufficient data in the patient chart.

### Statistical analysis

For the main analysis, two groups of children were compared: those who experienced a second seizure following their initial presentation and those who did not. Continuous variables were analyzed using Student’s *T*-tests or Mann–Whitney *U* tests, depending on the normality of their distribution as assessed by Shapiro–Wilk tests. For categorical variables, chi-square tests were utilized, with Fisher’s exact tests applied when expected cell frequencies were below five. The analysis encompassed a range of variables, including demographic characteristics such as age, gender, and population group. To account for multiple comparisons in post hoc analyses, *p*-values were adjusted using the Bonferroni correction method. All statistical analyses were conducted using RStudio version 2024.04.2 + 764, and a *p*-value < 0.05 was considered statistically significant for all tests.

We also compared hospitalized and non-hospitalized patients, focusing on demographic variables (age, gender, population sector), clinical factors (timing of seizure onset, duration of PED stay), historical elements (family history of febrile seizures, past febrile seizures), and mode of arrival to the PED. To identify predictors of hospitalization, we performed logistic regression analysis. Variables demonstrating significant associations in univariate analyses were incorporated into the multivariate model. The Hosmer–Lemeshow test was utilized to assess the goodness-of-fit of the logistic regression model.

## Results

Of 1496 pediatric patients initially screened for the study, 1404 met inclusion criteria; 62 children were excluded for having > 1 seizure prior to arrival, 26 for seizure duration > 15 min, and four because of underlying neurological conditions. Ten children, who presented to the PED with fever and who went on to experience a seizure in the department, were included in the 1404 patients. The clinical characteristics of children with single and multiple seizures were similar (Table 1).

**Table 1:** Demographic and clinical characteristics of children with a single febrile seizure versus those who experienced additional seizures during their hospital stay

	Single seizure (*N* = 1377)	Recurrent Seizures (*N* = 27)*	*P*-value
Age in months: *N* (%)			
12–24	728 (52.9%)	16 (59.3%)	0.563
24 +	649 (47.1%)	11 (40.7%)	
Gender distribution: *N* (%)			
Female	596 (43.3%)	12 (44.4%)	1
Male	781 (56.7%)	15 (55.6%)	
Ethnicity: *N* (%)			
Bedouin	679 (49.3%)	14 (51.9%)	0.853
Jew	696 (50.5%)	13 (48.1%)	
Other	2 (0.1%)	0	
History of previous febrile seizures: *N* (%)			
Yes	411 (29.8%)	9 (33.3%)	0.835
No	906 (65.8%)	18 (66.7%)	
Family history of febrile seizure: *N* (%)			
Yes	249 (18.1%)	4 (14.8%)	0.62
No	738 (53.6%)	18 (66.7%)	
Antipyretic medication at home: *N* (%)			
Yes	736 (53.4%)	17 (63.0%)	0.618
No	257 (18.7%)	4 (14.8%)	
Duration of fever prior to presentation in hours: *N* (%)			
< 3	183 (13.3%)	3 (11.1%)	0.583
3–12	243 (17.6%)	7 (25.9%)	
13–24	423 (30.7%)	6 (22.2%)	
> 24	377 (27.4%)	9 (33.3%)	
Time from seizure to arrival in hours: *N* (%)			
< 3	1128 (81.9%)	24 (88.9%)	0.305
3–12	125 (9.1%)	0	
> 12	34 (2.5%)	0	
Reported duration of seizure in minutes			
Mean (standard deviation)	4.54 (3.57)	4.52 (3.58)	0.936
Median (minimum, maximum)	4.00 (0.250, 15.0)	5.00 (0.100, 15.0)	
Fever at presentation: *N* (%)			
Yes	1140 (82.8%)	25 (92.6%)	0.297
No	237 (17.2%)	2 (7.4%)	
Maximal rectal temperature in Celsius: *N* (%)			
Low grade fever (37.3–38.0)	150 (11.0%)	1 (3.7%)	0.528
Moderate grade fever (38.1–39.0)	446 (32.4%)	10 (37.0%)	
High grade fever (39.1–41.0)	724 (52.65%)	15 (55.6%)	
Antipyretic medication in emergency department: *N* (%)			
Yes	760 (55.2%)	11 (40.7%)	0.409
No	606 (44.0%)	13 (48.1%)	
Time from seizure to discharge in hours: *N* (%)			
< 3	18 (1.3%)	0	< 0.001
3–6	297 (21.6%)	0	
6–12	591 (42.9%)	0	
13–24	65 (4.7%)	0	
> 24	406 (29.5%)	27 (100%)*	

*All 27 were admitted

Actual observation times varied: 315 patients (22.4%) were discharged within 6 h, 591 patients (42.1%) were observed for 6–12 h, and 471 patients (33.5%) required longer observation or admission. These variations reflected real-world clinical practice where rigid adherence to protocol timing was modified based on individual patient circumstances.

Table 2 compares the demographic and clinical characteristics of the 412 (29.3%) children who were hospitalized and those who were not. Hospitalization was more common among children who were younger (< 24 months [57.3%] versus > 24 months [51.2%], *p* = 0.045), Bedouin ([55.3%] versus Jewish [46.8%], *p* = 0.007), or who experienced a second seizure (*p* = 0.001). Recurrent seizure occurred in 27 (1.9%) children, all within 3 h of the initial seizure event, 22 in the PED, and five on the hospital ward.

**Table 2:** Demographic and clinical characteristics of children with febrile seizures by hospitalization status

	Not hospitalized (*N* = 992)	Hospitalized (*N* = 412)	*P*-value
Age in months: *N* (%)			
12–24	508 (51.2%)	236 (57.3%)	0.04
24 +	484 (48.8%)	176 (42.7%)	
Gender: *N* (%)			
Female	432 (43.5%)	176 (42.7%)	0.813
Male	560 (56.5%)	236 (57.3%)	
Ethnicity: *N* (%)			
Arab	465 (46.9%)	228 (55.3%)	0.007
Jew	525 (52.9%)	184 (44.7%)	
Other	2 (0.2%)	0 (0%)	
Site of seizure occurrence: *N* (%)			
Seizure before arrival at emergency department	992 (100%)	385 (93.4%)	< 0.001
Seizure in emergency department/ward*	0 (0%)	27 (6.6%)	
Time in emergency department in hours			
Mean (SD)	5.32 (3.15)	3.79 (3.74)	< 0.001
Median (min, max)	4.80 (0, 23.8)	2.98 (0.333, 23.0)	
Family history of febrile seizures: *N* (%)			
Yes	161 (24.0%)	92 (27.2%)	0.282
No	510 (76.0%)	246 (72.8%)	
History of past febrile seizures: *N* (%)			
Yes	304 (32.3%)	116 (28.8%)	0.222
No	637 (67.7%)	287 (71.2%)	
Method of arrival: *N* (%)			
Ambulance	440 (45.8%)	188 (47.4%)	0.632
Independent	520 (54.2%)	209 (52.6%)	
WBC value 10^3^/µL			
Mean (SD)	14.5 (5.71)	16.0 (6.74)	< 0.001
Median (min, max)	13.9 (2.46, 39.6)	15.0 (3.58, 39.8)	
Hemoglobin value g/dL			
Mean (SD)	11.6 (0.954)	11.5 (1.06)	0.045
Median (min, max)	11.6 (8.50, 16.2)	11.5 (7.90, 16.7)	
Sodium value mEq/L			
Mean (SD)	135 (2.25)	134 (2.61)	0.024
Median (min, max)	135 [128, 142]	135 [126, 143]	
Platelet value 10^3^/µL			
Mean (SD)	299 (85.7)	317 (96.7)	0.002
Median (min, max)	288 [53.0, 753]	306 [95.0, 733]	
CRP value mg/dL			
Mean (SD)	10.1 (18.3)	13.5 (26.9)	0.365
Median (min, max)	2.76 [0.0200, 155]	3.00 [0.0500, 217]	

*SD* standard deviation

*Min* minimum

*Max* maximum

*All recurrent seizures occurred within 3 h of the initial seizure; 22 in the PED and 5 on the hospital ward

Etiology of fever in hospitalized patients were 122 upper respiratory tract infection, 80 otitis media, 69 gastrointestinal infection, 34 tonsillitis, 29 pneumonia, 15 urinary tract infection, 1 cellulitis, 49 with other reported etiologies, and 13 with no conclusive diagnoses. Six hospitalized patients were still hospitalized 48 h after admission but none of these experienced seizure recurrence and the prolonged hospitalization was unrelated to the febrile seizure episode.

Logistic regression examined ethnicity, age in months, and total time from seizure to discharge as predictors of hospitalization; being Jewish was associated with lower odds of hospitalization compared to being Bedouin (odds ratio = 0.7, 95%; confidence interval [0.55, 0.88], *p* = 0.002). Age showed a small but significant negative association with hospitalization, with each additional month of age associated with slightly lower odds of hospitalization (odds ratio = 0.99; 95% confidence interval [0.98, 0.99], *p* = 0.033). Similarly, longer total time from seizure to discharge was associated with decreased odds of hospitalization (odds ratio = 0.91, 95%; confidence interval [0.87, 0.94], *p* < 0.001).

Most patients underwent a complete blood count (*n* = 1395, 99%) and blood chemistry panel (*n* = 1375, 97.9%) with no significant differences between hospitalized and non-hospitalized children in mean levels of hemoglobin, white blood cell count, platelet count, sodium levels, or C-reactive protein (data not shown).

In our study cohort, no patients were readmitted after discharge from the PED or the wards due to recurrent seizures or complications related to seizures. No children required admission to the intensive care unit, and our cohort experienced no fatalities.

## Discussion

In our cohort of 1404 children aged 12 months to 5 years with a febrile seizure, 99.1% could finally be diagnosed with a SFS. Our experience suggests that even though the criterion regarding non-recurrence during the ensuing 24 h to define SFS has perforce not been met, children could be safely discharged following a febrile seizure, provided also that the clinical source of their fever is benign. Beyond monitoring for seizure recurrence, observation in the PED serves an important role in caregiver reassurance and parental education. Providing families with guidance and reassurance is essential for ensuring safe discharge and empowering them to manage febrile seizures at home. Our findings indicate that, because almost all recurrences occur within the first 3 h, these goals can usually be achieved during a relatively short observation period, avoiding the need for prolonged mandatory stays.

A meta-analysis by Henry et al. [14] reported a high recurrence rate of 17%; however, their data included patients with known neurological conditions and/or other risk factors for seizures, in addition to reflecting recurrence within a 24-h period. Although our low recurrence rate of 1.9% may be because we only followed patients for 6 h, it may also be because we only included children who fulfilled all other criteria for SFS. Although certain variables, such as fever at presentation, occurred more frequently in recurrent cases, these differences did not reach statistical significance. Given the small number of recurrent events (*n* = 27), our study lacks the power to definitively identify independent risk factors for recurrence, and our findings should be considered hypothesis-generating. However, we still maintain that our low overall recurrence rate and the fact that almost all recurrences occurred within the first 3 h contribute clinically meaningful data to the discussion on observation protocols.

Our study did not show a relationship between duration of fever prior to occurrence of febrile seizure and seizure recurrence. Although some studies have claimed that a shorter lead-time is a risk factor for subsequent febrile seizures [9, 23, 24], others did not confirm this [25]. There is also some debate over the impact of lead-time on multiple seizures during a single fever episode [16, 26]. While in our cohort a prior history of febrile seizures or a family history of febrile seizures were not associated with an increased risk of short-term recurrence (Table 1), caution is advised when applying these results to “gray zone” cases, as clinical decision-making may differ in practice.

We did not observe any correlation between antipyretic use and seizure recurrence. The potential for antipyretics to reduce the likelihood of febrile seizure recurrence during the same illness remains controversial. Murata et al. found that children receiving scheduled acetaminophen had a 14.4% lower incidence of additional seizures compared to those who received no treatment [12], while a study by Schnaiderman et al. showed no difference in seizure recurrence between children given scheduled versus intermittent acetaminophen [27].

There is an acknowledged relationship between lower temperature at seizure onset and seizure recurrence > 24 h after the index febrile seizures [28], potentially reflecting a lower seizure threshold in these patients. However, the significance of fever as a predictor of recurrent febrile seizures within 24 h remains controversial, with varying conclusions reported in the literature. In a recent prospective study involving 109 children, Kubota et al. [29] found that body temperature < 39.2 °C was significantly associated with recurrence (*p* = 0.02). Similar findings were noted in their earlier retrospective pilot study of 132 children [26]. Furthermore, additional studies have demonstrated that those presenting with an initial temperature < 39 °C were more prone to recurrence than those with higher temperatures [30]. In contrast, consistent with other studies such as Jeong et al. [31], we did not observe any association between body temperature and recurrence of seizure.

The specific role of gender in influencing the risk of recurrence within the first 24 h after the initial febrile seizure has been examined in a number of studies with varying results. Okubo et al. [32] reported males having a 1.75 times higher rate of febrile seizure readmission than girls. A large-scale study by Berg et al. [33] found that while boys might show a slight predominance in febrile seizure incidence, gender by itself was not a significant independent factor for predicting recurrent seizures within 24 h. Similarly, in our study, male sex alone was not a strong or consistent predictor of seizure recurrence within the 6 h following the index febrile seizure.

Although certain ethnic groups exhibit different overall frequencies of febrile seizures [16], current evidence does not consistently identify ethnicity as a primary determinant of short-term recurrence within the same febrile illness. The population treated in our PED consists mainly of Jewish and Bedouin children. Our cohort showed no differences between these two population groups. Future studies with diverse populations, accounting for healthcare practices and genetic background, may offer more clarity on whether ethnicity plays any direct, independent role in the short-term recurrence of febrile seizures.

While many studies have established that family history is an important risk factor for febrile seizure recurrence [23, 25, 34], the literature is more mixed regarding its impact on immediate or same-illness recurrence [31, 35]. In our cohort, there was no association between family history of febrile seizures and recurrence. Furthermore, although a prior history of febrile seizures is a known risk factor for future episodes [6], in our cohort, it did not independently predict seizure recurrence within the same febrile illness.

The 45.8% ambulance utilization rate reflects our mixed catchment area where many families opt for private vehicle transport due to geographic dispersion and familiarity with direct routes to our facility.

## Study limitations

This is a single-center study, relying on retrospective data from medical records; as such, incomplete or inconsistent documentation may have affected accuracy of data. Data on fever onset, febrile illness, seizure duration, and time of seizure prior to emergency department arrival relied on parental reporting, which may be subject to recall bias or inaccuracies. Findings may not be generalizable to populations with different demographics or healthcare systems. The study focused on recurrence during the PED admission, possibly missing seizures that occurred after discharge. However, since Soroka Medical Center is the only pediatric emergency facility in the region, it is highly likely that most recurrent seizure events would have been captured in our data, and any missed cases are expected to be very rare. The relatively small number of recurrent seizures limits the ability to detect subtle associations between clinical variables and recurrence. Larger, prospective, multicenter studies will be necessary to confirm or refute these potential trends.

## Conclusions

Provided that the etiology of fever is established as being non-serious, our findings suggest that children who have experienced a febrile convulsion fulfilling all the criteria of SFS aside from the requirement of not suffering another seizure in the 24 h after the index seizure may be considered for early discharge from the PED upon normalization of their neurological status. In certain situations, such as when children have underlying conditions, limited access to healthcare, or significant parental anxiety, a short period of observation may be considered based on clinical judgment, although this recommendation is not directly supported by the data from this study.

## Data Availability

The data that support the findings of this study are not openly available due to reasons of sensitivity and are available from the corresponding author upon reasonable request. Data are located in controlled access data storage at Soroka Medical center.

## Abbreviations

PED: Pediatric emergency department
SFS: Simple febrile seizures
